# Enterococcal bacteriophage: A survey of the tail associated lysin landscape

**DOI:** 10.1016/j.virusres.2023.199073

**Published:** 2023-02-22

**Authors:** Alhassan M. Alrafaie, Graham P. Stafford

**Affiliations:** a Integrated BioSciences, School of Clinical Dentistry, University of Sheffield, Sheffield, United Kingdom; b Department of Medical Laboratory Sciences, College of Applied Medical Sciences in Al-Kharj, Prince Sattam Bin Abdulaziz University, Al-Kharj 11942, Saudi Arabia

**Keywords:** Bacteriophages, Prophage, *Enterococcus*, Tail-associated lysin

## Abstract

Bacteriophages are viruses that exclusively infect bacteria which require local degradation of cell barriers. This degradation is accomplished by various lysins located mainly within the phage tail structure. In this paper we surveyed and analysed the genomes of 506 isolated bacteriophage and prophage infecting or harboured within the genomes of the medically important *Enterococcus faecalis* and *faecium*. We highlight and characterise the major features of the genomes of phage in the morphological groups podovirus, siphovirus and myovirus, and explore their categorisation according to the new ICTV classifications, with a focus on putative extracellular lysins chiefly within tail modules. Our analysis reveals a range of potential cell-wall targeting enzyme domains that are part of tail, tape measure or other predicted base structures of these phages or prophages. These largely fall into protein domains targeting pentapeptide or glycosidic linkages within peptidoglycan but also potentially the enterococcal polysaccharide antigen (EPA) and wall teichoic acids of these species (i.e., Pectinesterases and Phosphodiesterases). Notably, there is a great variety of domain architectures that reveal the diversity of evolutionary solutions to attack the Enterococcus cell wall. Despite this variety, most phage and prophage possess a putative endopeptidase (70%), reflecting the ubiquity of this cell surface barrier. We also identified a predicted lytic transglycosylase domain belonging to the glycosyl hydrolase (GH) family 23 and present exclusively within tape measure proteins. Our data also reveal distinct features of the genomes of podo-, sipho- and myo-type viruses that most likely relate to their size and complexity. Overall, we lay a foundation for expression of recombinant TAL proteins and engineering of enterococcal and other phage that will be invaluable for researchers in this field.

## Highlights

• 506 enterococcal phage and prophage genomes were scanned for tail module lysins.
• Identified lysins target cell wall, teichoic acids and enterococcal polysaccharide antigen.
• Most common identified lysin was endopeptidase in both phage and prophage genomes.
• Lytic transglycosylase lysins possess unique motifs and locate in tape measure proteins.
• This study provides a rich foundation for phage engineering and TAL recombinant expression.

## 1 Introduction

The antibiotic resistance crisis is a major global health threat, with a predicted potential 10 million deaths annually by 2050 if unchecked (O’Neil, 2016). One particular bacterial genus that has contributed to this crisis is the enterococci, Gram-positive bacteria that normally inhabit the gastro-intestinal tract (GIT) of humans as well as other animals such as reptiles, fish and insects (Bondi et al., 2020). GIT colonization by enterococci is achieved through multiple factors such as genome plasticity, nutritional adaption and antimicrobial production and resistance (Banla et al., 2019). As a pathogen, they are commonly involved in human wound infections (Bowler et al., 2001), bacteraemia, endocarditis, urinary tract infection (UTI) (Ben Braïek and Smaoui, 2019) as well as recalcitrant endodontic dental infections (Love, 2001). Until 1984 enterococci were classified as *Streptococcus* but were reclassified into their own genus, with the two main species associated with human diseases being *Enterococcus faecalis* and *faecium* (Schleifer and Kilpper-Balz, 1984). These organisms the first to become resistant to the glycopeptide antibiotic of last resort Vancomycin in 1986- classed as vancomycin-resistant enterococci (VRE) (Uttley et al., 1988). These have now spread worldwide and have been recognized by the World Health Organisation (WHO) as high priority to be targeted and investigated for new antimicrobials (Willyard, 2017).

One area of interest that has seen a resurgence in the context of fighting multidrug resistant pathogens is in the use of naturally occurring antibacterial viruses known as bacteriophage, often simply called phage (Dion et al., 2020). Phages were discovered in the early 20th century by Frederick Twort in 1915 (Twort, 1915) and Felix d'Herelle in 1917 (Terms, 2011) and are likely to be the most abundant biological entities on our planet with estimates as high as 10^31^ globally at any one time (Suttle, 2005). Phages are thus found almost everywhere in our environment, with abundant phage found in soil, wastewater sewage, ocean sediment and the human body.

Much like viruses that infect humans, multiple infectious lifecycles exist with the two major ones being designated lytic and lysogenic lifecycles, with temperate phages being those that can follow both lifecycles. The first step in phage infection is the adsorption of phage to the host surface, followed by genome injection into the bacterial cytoplasm. At this stage, lytic phages take over the infected cell to synthesise virions before a critical load is reached in the cell before virions are released and the bacterial cells lysed from the inside-out- using phage (endo)lysins (Abdelrahman et al., 2021). For temperate phages, the phage genome is either integrated into the bacterial genome (known as a prophage) or remains dormant as a plasmid in the bacterial cytoplasm. The phage genome can be induced to allow the temperate phage to then undergo a lytic cycle. For phage therapy, lytic phages are the first choice since they obligatorily lyse their hosts.

During the phage lifecycle, phage must first adhere to the target bacterial cell surface but must also present their DNA-injection machinery in close enough proximity to the host cell membrane. In some cases, this is a particular challenge as the bacterial strain might be coated with capsular material, in the case of enterococci these include enterococcal polysaccharide antigen (EPA) (Guerardel et al., 2020) as well as peptidoglycan and teichoic acids (Silhavy et al., 2010). As a result phage often contain lysins that are often associated with components of the phage virion structural proteins, such as terminal tail structures or tail tape measure proteins. Hence these are often known as Virion-associated lysins (VAL) or Tail-associated lysins (TAL), we will use the latter term here.

**Fig. 1:**
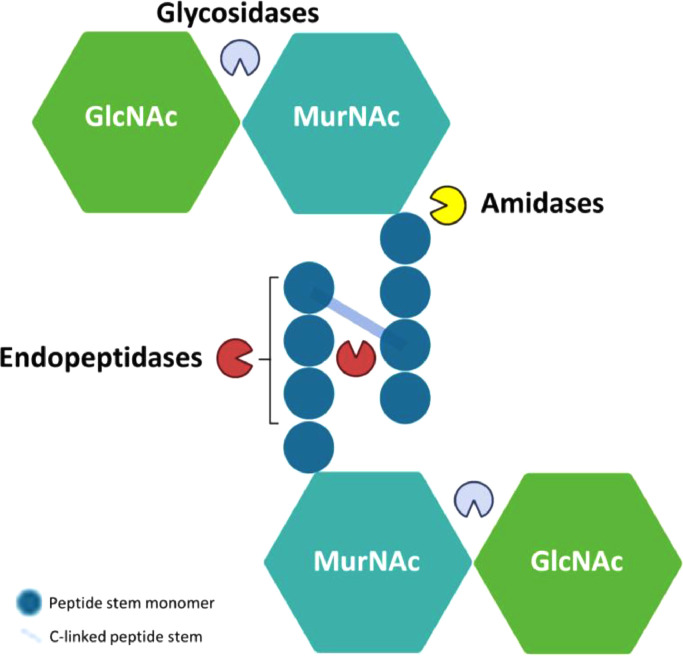
Schematic representation of the bacterial peptidoglycan structure showing target locations of Glycosidases (grey), Amidases (yellow) and Endopeptidases (red). MurNAc: N-acetylmuramic acids GlcNAc: N-acetylglucosamine. This figure was created with Biorender.com.

Based on the mode of action, TALs can be classified into three main classes: glycosidases, amidases and endopeptidases (Latka et al., 2017) (Fig. 1). The Glycosidases often target the β−1,4 glycosidic bonds in the sugar moiety of peptidoglycan and are divided into three subtypes. First, N-acetyl- β-d-muramidases that cleave the link between N-acetylmuramic acids (MurNAc) and N-acetylglucosamines (GlcNAc). Secondly, N-acetyl-β-d-glucosidases target the bond between GlcNAc and MurNAc. The third subtype are Lytic transglycosylases which require water molecules for lysing MurNAc-GlcNAc linkages. The second class are amidases (N-acetylmuramoyl-l-alanine amidases) that cleave the bond between MurNAc and the first amino acid (L-alanine) in the peptide stem. Finally, the third class are endopeptidases which cleave the peptide bond either within the interpeptide bridge or stem peptide of peptidoglycan (Elbreki et al., 2014).

In this study we sought to explore the potential TAL landscape of sequenced enterococcal phage, as well as predicted prophage from the genomes of sequenced enterococcal strains of *E. faecalis* and *E. faecium* with a view to potentially using this information to design novel antimicrobials in the future. These studies are made possible since phage genomes are efficiently ordered with functional modules clustered in their genomes (Moura de Sousa et al., 2021). Examples of these modules include packaging, head, tail and lysis modules. Of the over 20,000 sequenced phage genomes, to date 162 infect enterococcal strains (October 2022), but no investigations of the TAL landscape for these have been undertaken to our knowledge. Therefore, we investigated TALs in both enterococcal phage and prophage genomes which resulted in identifying various TALs targeting different bacterial layers. Our work also highlights previously unreported unique features for several enterococcal TALs.

## 2 Methods

### 2.1 Phage and prophage genomes

One hundred complete enterococcal phage DNA genomes available on the NCBI GenBank database were obtained as Genbank and Fasta sequences (up to 11/10/2020). The search for these genomes was done on the NCBI virus portal by using “bacteriophage” for virus choice, “Genbank” sequence type, “complete” for genome sequence and “Enterococcus” for host. The GenBank accession numbers of these genomes are included in supplementary file 1. For prophage genomes 203 complete *E. faecalis* and *E. faecium* bacterial genomes available on the NCBI GenBank database were obtained (up to 10–10–2020), accession numbers are included in supplementary files 2 and 3. The online web server PHASTER (Arndt et al., 2016) was used to identify putative intact prophages. All phage and prophage genomes were re-annotated to ensure annotation consistency using RASTtk (new version of Phage Rapid Annotation using Subsystem Technology (RAST) pipeline (Brettin et al., 2015).

### 2.2 TAL identification and analysis

The tail module was identified between the head and lysis modules in most of the phage and prophage genomes based on RASTtk annotation. All tail proteins were checked for TALs using Pfam and NCBI conserved domains (CDD) databases. Structural analysis was also performed using the PHYRE2 webserver (Kelley et al., 2015). SnapGene (v 5.3.2) and Artemis (Carver et al., 2012) were used for genome visualisation. The identified TAL proteins were aligned using ClustalW (genome.jp) and MultAlin webservers (Corpet, 1988). Phylogenetic trees were constructed using FastTree (genome.jp) and visualised using the ITOL online website (Letunic and Bork, 2021) (https://itol.embl.de/). To check putative peptidase classifications, the MEROPS database was employed (http://www.ebi.ac.uk/merops/) (Rawlings et al., 2018) while the CAZy (Carbohydrate Active Enzymes) database (CAZy; http://www.cazy.org) (Lombard et al., 2014) was used for predicted Glycoenzymes. Genome size analysis was performed using GraphPad Prism version 7,San Diego, California USA, www.graphpad.com.

## 3 Results and discussion

Using the NCBI virus portal, the genome sequences of 100 phage targeting either *Enterococcus faecalis* or *Enterococcus faecium* were collected for analysis. These comprised 86 phages isolated using *E. faecalis* strains while 10 phages were isolated using *E. faecium* strains. For predicted prophages, a total of 203 *E. faecalis* & *E. faecium* complete genomes were scanned using the PHASTER prediction tool with the default parameters set for classification of “intact” prophage set at (>90%), “questionable“ (scoring 70–90%) and “incomplete” (scoring<70%). In this study we focused on the intact prophages as these have the highest confidence level to maintain a full set of functional modules and allow tail module identification. The PHASTER searches revealed 406 intact prophages in both *E. faecalis* and *E. faecium* bacterial genomes, meaning that in total this study examined 506 phage and prophage genomes.

### 3.1 Isolated phage genomes for *Enterococcus* are members of a range of viral classes

**Fig. 2:**
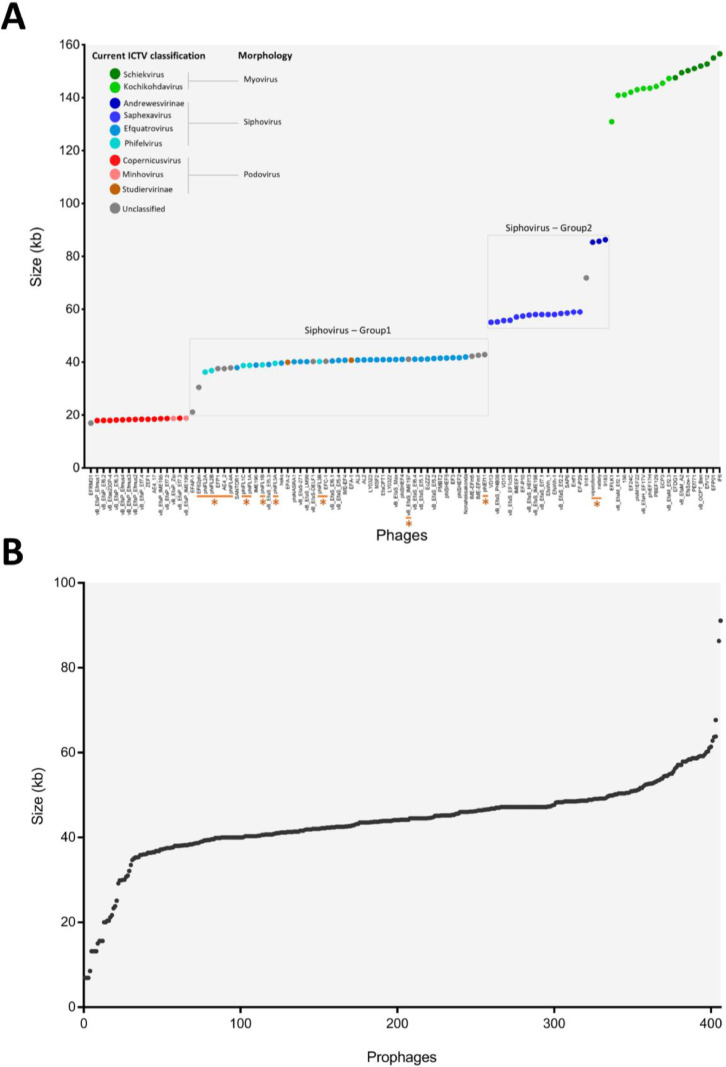
(A) 100 enterococcal phage genomes were plotted against genome size. The genomes are labelled in accordance with the new ICTV classification as follow: Schiekvirus (dark green), Kochikohdavirus (light green), Andrewesvirinae (dark blue), Saphexavirus (blue), Efquatrovirus (Azure), Phifelvirus (sky), Copernicusvirus (red), Minhovirus (orange), Studiervirinae (brown),. The grey colour indicates unclassified genomes regarding the current ICTV classification and further details are included in the supplementary file1. Phage morphologies are also included according to the ICTV classification. Temperate phages are underlined and labelled with asterisks. (B) 406 intact prophage genomes were plotted against genome size. The genomes are in ascending order in both figures.

The 100 isolated phage genomes showed a variation in size from 16.9 to 156.5 kb (Fig. 2A). Our analysis showed that the phage genomes can be categorised into three main groups based on genome size and phage virion morphology. Phages with small genomes (<30.5 kb) are generally podoviruses (Rountreeviridae), medium sized genomes (31–86.3 kb) siphoviruses (Efquatrovirus, Phifelvirus, Saphexavirus and Andrewesvirinae), and large genomes of over 130 kb myoviruses (Herelleviridae) (Fig. 2A). There were some exceptions, for example, the smallest *Enterococcus* phage EFRM31 (16.9 kb) is a siphovirus (unclassified according the current ICTV classification) with an isometric head and long non-contractile tail (206 nm tail length), whose genome has 35 predicted ORFs (Open Reading Frames) (Fard et al., 2010). Other examples of note within the podoviruses (Autographiviridae) are the *E. faecalis* phages EFA-1 (40.7 kb) and EFA-2 (39.9 kb) which have higher GC contents and number of ORFs (EFA-1 is 50.14%, 52 ORFs and EFA-2 is 48.55%, 49 ORFs) compared to the average value for enterococcal podoviruses (Autographiviridae and Sarlesvirinae) analysed in this study (35.1%, 30 ORFs).

Regarding morphology, all the 100 enterococcal phages are predicted morphologically to be either podoviruses (short tailed), siphoviruses (long non-contractile tail) or myoviruses (contractile tail) based on database entries. While the morphological categorisation of podo- myo- and sipho-virus has been widely used for many years, the recent increase in genomic information has identified a number of differences and allowed continual improvement of phage taxonomy (Turner et al., 2021). However, we will in some places use the commonly used morphological terms to simplify discussions. All of the 18 small genomed predicted podoviruses are classified as Copernicusvirus or Minhovirus within the Rountreeviridae or belong to Autographiviridae according to the new ICTV classifications and have a genome size of 17.9 to 40.7 kb (Fig. 2A) (Turner et al., 2021). The number of ORFs encoded in these genomes ranged from 22 to 52 with an average of 30.

Siphoviruses make up 64% of the isolated phage with genomes ranging from 16.9 to 86.3 kb. Based on the genome size and TAL analysis, siphoviruses can be classified into two groups: group-1 (21–43 kb, Efquatroviruses or Phifelviruses) and group-2 (55–86 kb, Saphexavirus or Andrewesvirinae) (Fig. 2A). The average number of predicted ORFs in group-1 is 62 while this is 104 for Group-2. Lastly, we analysed 18 myovirus-type genomes (Herelleviridae, Schiekvirus or Kochikohdavirus) where the genomes varied from 130.9 to 156.5 kb (average 146.5 kb). The new classifications are further supported by these data since the genome sizes alone can indicate likely species membership according to our data.

Unsurprisingly, a positive correlation was seen between phage genome size and the number of ORFs with the small podoviruses having the lowest number of ORFs and the largest genomes (myoviruses) the highest ORFs number (Fig. S1A). The number of tRNAs also shows a positive correlation with the genome size, with podovirus genomes having no tRNAs while larger genomes of siphoviruses and myoviruses contain several putative tRNA genes (Fig. S1B). In contrast, there is no clear correlation between the genome size and GC content (Fig. S1C).

Of the 100 enterococcal phage genomes we identified several temperate phages, based on the presence of integrase and repressor genes that are necessary for phage integration and maintenance during the lysogenic cycle into the bacterial genome. In our data, 16% of viruses are likely to be temperate, as they contain integrase and/or repressor genes (Fig. 2A). Of these temperate phages, only one is reported to be pseudotemperate, and was identified in the genome of *E. faecalis* 62 and contains a toxin-antitoxin system (Brede et al., 2011).

### 3.2 Enterococcal prophages

For prophages, only predicted intact prophage genomes were chosen and analysed. In our study, a total of 406 putative intact prophages (93 from *E. faecalis* & 313 from *E. faecium* genomes) were identified, with the most in one genome being five from 203 genomes that were scanned. These showed large variation in the predicted genome size (6.9–91.1 kb) (Fig. 2B), with the smallest prophage containing 10 ORFs and the largest 121 ORFs with an average GC content of 35.9%. It is worth mentioning here that not all identified intact prophage genomes possessed all necessary genes to complete phage lifecycle indicating a limitation of the PHASTER webserver. Our analysis also showed a positive correlation between the number of ORFs and prophage genomes size (Fig. S2A). The analysis of the number of tRNA genes showed no correlation (Fig. S2B).

### 3.3 Five types of predicted tail associated lysins exist in enterococcal phage genomes

**Fig. 3:**
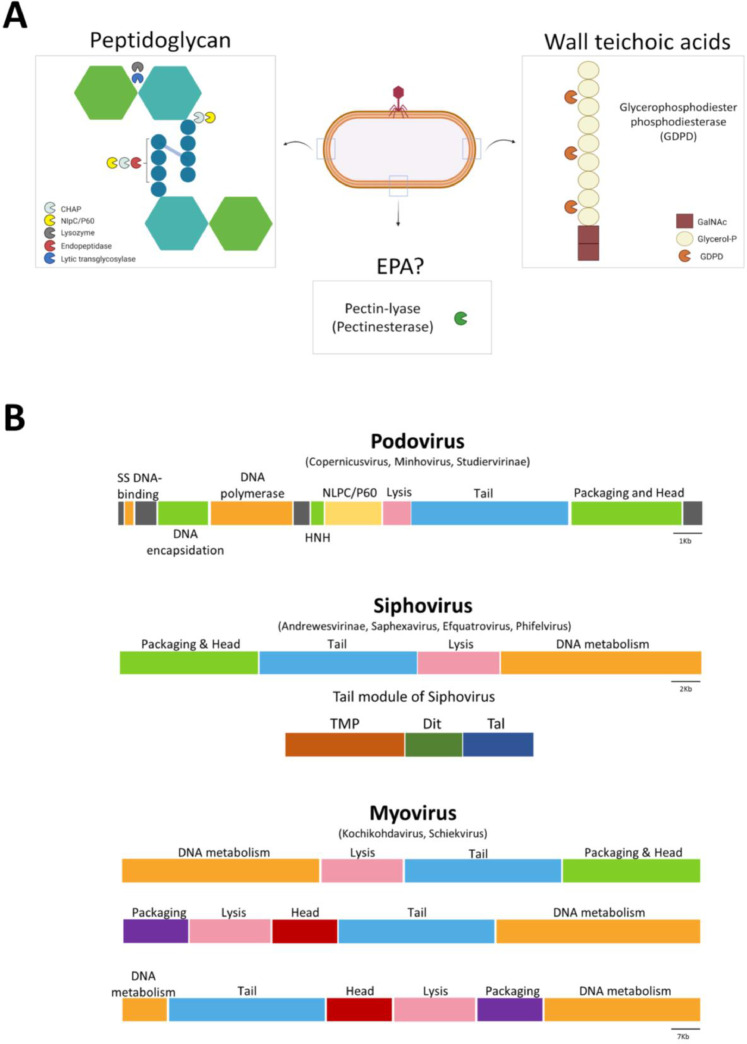
(A) Schematic representation of the identified lysins and their targets in this study. This part was created with Biorender.com. (B) The order of the Functional modules of the enterococcal Phage genomes. Modules and specific genes are coloured as follow: Green= DNA packaging and head, Red= Head alone, Purple= Packaging alone, Blue= Tail, Pink= lysis, Orange= DNA metabolism. Of note, podovirus general genome organisation contains some labelled gene like yellow= NLPC/P60 gene. HNH stands for homing endonuclease. The general scheme of the siphovirus tail module is also drawn Brown=TMP, Dark green= Dit and Dark blue=Tal. The new ICTV classification is also indicated regarding each phage morphology.

Phage genomes are generally organised in modules where related functional genes are grouped together such as packaging, head, tail and lysis functions. For example, the tail module of siphovirus type phage is considered to generally comprise of three main genes in the following order: “Tape measure protein”- TMP, “Distal tail protein”- Dit and “Tail associated Lysin”- Tal (Goulet et al., 2020) (Fig. 3B). In this study, the term “TAL” means any lysin in the tail module while “Tal” is referring to the third gene in the siphovirus tail unit.

The TMP is usually one of the longest genes in phage genomes, and plays a role in controlling tail length, with the length of its translated protein approximately indicating the length of the phage tail (1 amino acid= 0.15 nm) (Mahony et al., 2016). The TMP also helps facilitate genome ejection toward the bacterial cytoplasm, although mechanistic details are unclear (Mahony et al., 2016). This is evidenced by identifying domains with potential cell wall degrading function in TMPs as well as DNA-binding domains (Piuri and Hatfull, 2006; Stockdale et al., 2013). TMPs are thought to be located in the lumen of the tail and interact with termination and initiation proteins as well as the polymeric Major Tail Protein (MTP)(Cornelissen et al., 2016; Kizziah et al., 2020).

The Dit is part of the baseplate and connects the tail with the tail tip as well as providing in some cases the site of attachment for a RBP “receptor-binding protein”- which may be housed on a fibrous protein (Kizziah et al., 2020). The RBPs are responsible for the specific recognition of bacterial receptors that may include outer membrane proteins, bacterial capsule, teichoic acids, pili and flagella (Bertozzi Silva et al., 2016; Letarov and Kulikov, 2017).

In this study we assessed all genes in the putative tail modules (not only the putative Tal) for the presence of lysin- like domains, so as not to exclude any that might be associated directly with TMPs, RBP or tail fibres since many lysins used by phages in the first steps of phage infection are associated with the tail and baseplate structure (Latka et al., 2017). After obtaining and reannotating the enterococcal phage and prophage genomes, all the predicted tail genes were scanned for the presence of predicted lysin domains by using Pfam, the NCBI Domain database and the Phyre2 webserver. As a result, multiple types of lysins were identified in both phage and prophage genomes (Table 1) (Fig. 3A).

**Table 1:** Summary of predicted lytic domains associated mainly with the tail module of our study set.

	Domain	Activity	# Sequences (% total 544)
Lysins	Endopeptidase	Endopeptidase	383 (70.4%)
	Lytic transglycosylase	Lytic transglycosylase	98 (18.0%)
	NLPC/P60	Endopeptidase or Amidase	34 (6.2%)
	Glycerophosphodiester phosphodiesterase (GDPD)	Phosphodiesterase	22 (4.0%)
	Pectinesterase	Pectinesterase	7 (1.3%)

Our analysis showed that presence of a predicted endopeptidase is the most common lysin associated with tail proteins (70.4%), while lytic transglycosylase domains were present in 18.0% of the total identified lytic proteins. These two types of lysins are preferentially carried by phage infecting Gram-positive bacteria (São-José, 2018). Other proteins were also observed to carry other potential lysins, namely peptidases of the NLPC/P60 family (6.2%), GDPD (4.0%) and lastly Pectinesterases (1.3%). Each one of these lysins are further discussed in the following sections.

#### 3.3.1 Enterococcal endopeptidases (TAEP) display a range of domain architectures

**Fig. 4:**
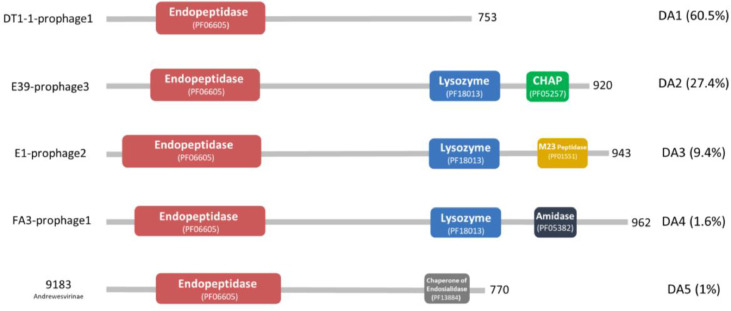
Domain architectures of TAEP proteins based on Pfam. Five DAs are shown with coloured domains. Red= endopeptidase, Blue= lysozyme, Green= CHAP, Yellow= M23 peptidase, Dark blue= amidase, Grey= chaperone of endosialidase. The left side contains the phage or prophage's name and ICTV classification while the right side contains the DA type and its abundance in percentage. The length of the protein is also indicated on the right side.

The **t**ail proteins **a**ssociated with **e**ndo**p**eptidase activity (TAEP) were identified in both phage and prophage genomes. We identified 383 TAEP proteins via homology with predicted phage endopeptidase domains (Pfam: PF06605). These TAEP proteins were then assessed for domain architectures (DA). This revealed 5 different groups (DA-EP), all containing a phage endopeptidase domain (Pfam: PF06605) located at the N-terminal end of the predicted protein (Fig. 4). These domains are all found in the Tal position (i.e. TMP-Dit-Tal), although it is not clear if they have an endopeptidase activity themselves or are involved in forming active complexes or act in a structural manner. Catalytically, endopeptidases target peptide bonds within peptidoglycan- either in the peptide stem or cross-bridge. Of our identified TAEP proteins 60.5% are within the DA1 architecture group and only contain an endopeptidase domain (Fig. 4). This has also been noticed previously as most Tal proteins harboured a single lysin (Latka et al., 2017). To further analyse the endopeptidase domains, the MEROPS database was used to check the peptidase family of these sequences. To do this, three representative sequences from each DA were screened against the MEROPS_scan dataset, resulting in highlighting two types (M23B and C104) with high E-value (<10^−10^).

The other DAs showed various lysin domains in addition to the endopeptidase domain. In DA2,3 and 4 a predicted lysozyme domain (Pfam: PF18013) was identified which is a structural homologue of a cell wall degrading enzyme in the bacteriophage phi29 tail (established using Phyre2 analysis) (Xiang et al., 2008). Besides the phage tail lysozyme, DA2 contains a CHAP domain (cysteine, histidine-dependant amidohydrolases/peptidase), while DA3 harbours a peptidase M23 domain (thought to target the peptide bonds in the peptidoglycan layer (Vermassen et al., 2019). DA4 also contains predicted amidase domains likely attacking the amide bond between MurNAc and the first amino acid l-alanine leading to separation of the glycan and peptide units. Finally, DA5 contains a domain with homology to endosialidase chaperones. Of these domains, all have been associated with cell wall degradation or in the case of DA5- stabilisation of other catalytic domains. For example, CHAP domains (Pfam:PF05257) have been shown to act as endopeptidases (e.g. LysK CHAP) (Becker et al., 2009) or amidases Proença et al., 2012). The Peptidase M23 domains (Pfam: PF01551) in DA3 are located at the C-terminal region as well as the predicted amidase domains (Pfam: PF05382) in DA4. The chaperone of endosialidase in DA5 has shown to facilitate the folding and assembly of endosialidases and other phage proteins as well, as it is eventually cleaved off to ensure the stability of the native protein (Schwarzer et al., 2007).

#### 3.3.2 Enterococcal new lipoprotein C/Protein of 60-kDa (NLPC/P60) are grouped based on phage morphology and genomic classification

It is known that many phage proteins contain domains belonging to the NLPC/P60 family (New Lipoprotein C/Protein of 60-kDa). The NLPC/P60 family is a large group of papain-like cysteine proteases present in bacteria, like *Escherichia coli* (NLPC) and *Listeria monocytogenes* p60 (Anantharaman and Aravind, 2003). The members of the NLPC/P60 family can have (endo)peptidase as well as other activities such as amidase, transglutaminases and acetyltransferase and often contain a conserved catalytic N-terminal cysteine and C-terminal Histidine residue (Anantharaman and Aravind, 2003). In bacteria the NLPC/P60 peptidases are likely to be involved in the bacterial cell cycle and morphogenesis by hydrolysing the peptidoglycan layer while in phage they likely aid in local peptidoglycan degradation and hence promoting genome injection (Fukushima et al., 2018; Griffin et al., 2022).

**Fig. 5:**
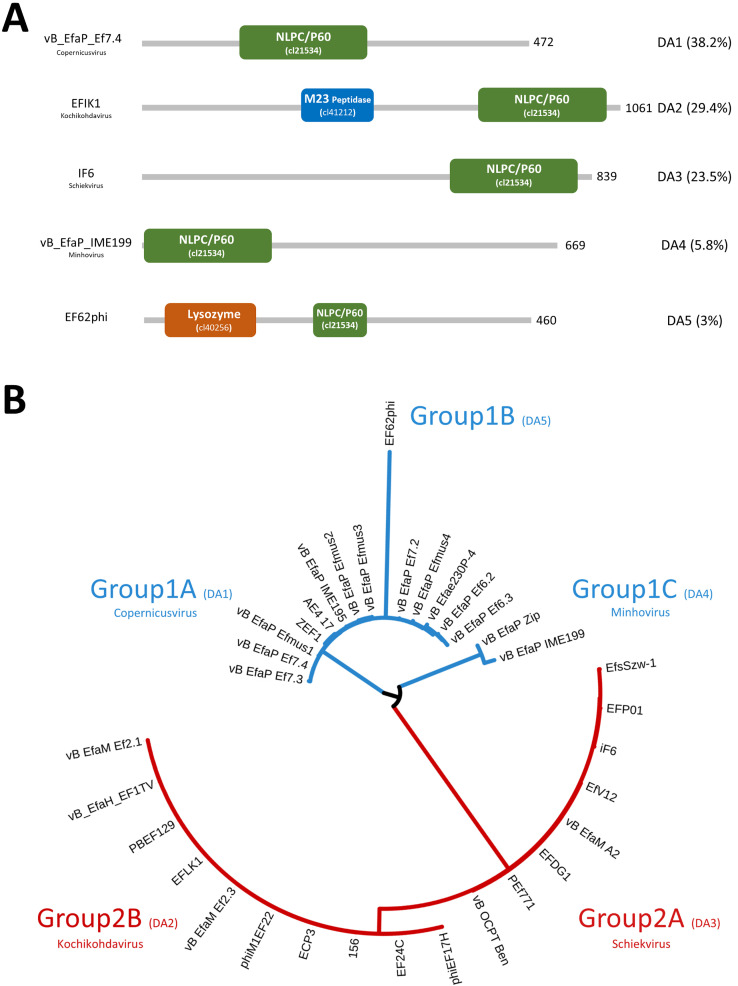
(A) Domain architectures of NLPC/P60 containing proteins. Five DA are shown with coloured domains. Green= NLPC/P60, Blue= M23 peptidase, Orange= lysozyme. The domain type and abundance (%) are indicated on the right side while phage or prophage name and the ICTV classification is on the left side. The length of the protein is also indicated. (B) a phylogenetic tree of all identified NLPC/P60 containing proteins show two main groups based on phage genomic classification and morphology: sequences from podoviruses labelled Blue while myoviruses labelled red. The tree was constructed using FastTree and visualised using the ITOL online website.

Our analysis identified 34 tail proteins that appear to be within the NLPC/P60 family (accession no.cl21534). Based on a phylogenetic tree made from amino acid alignments, these 34 sequences are classified into two main groups (Fig. 5B). Group1 includes sequences from podovirus-type genera while group 2 are from predicted myovirus subtypes.

Of these, Group 1 can be divided into three subgroups: 1A (Genus: Copernicusvirus) consists of 13 sequences which represent DA1 (Fig. 5A). The 1B subgroup includes only one sequence (EF62phi) which is clearly diverged from the 1A sequences and contains an additional lysozyme domain as shown in DA2. The 1C subgroup (Genus: Minhovirus) consists of sequences from podoviruses that were isolated using *E. faecium* strains in contrast to subgroups 1A and 1B (host strains are *E. faecalis*) as well as the NLPC/P60 domain at the N-terminal region as represented in DA4 (Fig. 5A).This may indicate differences in substrate specificity given the differing crosslinks between these spp. (Lys-Ala-Ala-for *E. faecalis* and Lys-Asx for *E. faecium*) and is the subject of current work in our lab (Arbeloa et al., 2004)

For group 2, these NLPC/P60-containing proteins were all found in myoviruses (Herelleviridae). This group can also be further subdivided based on the phylogenetic tree and domain architecture into subgroups: 2A and 2B (Fig. 5B). The 2A subgroup (Genus: Schiekvirus) contains only the NLPC/P60 domain, while the 2B subgroup (Genus: Kochikohdavirus) contain an additional M23 peptidase domain besides the NLPC/P60 domain.

Overall, we have revealed the presence of a range of lysins in tail modules of enterococcal phage, many of which that contain multiple lysin domains can be attributed to the need to degrade the various cell wall components and might work together to facilitate entry into enterococci. Finally, our data seem to indicate that the putative lysins group according to viral genus classification, suggesting shared function, but whether this relates to host-range is yet to be established.

#### 3.3.3 Tailed enterococcal phage contain tail tape measure proteins with lytic transglycosylase domains (TMP-LT)

**Fig. 6:**
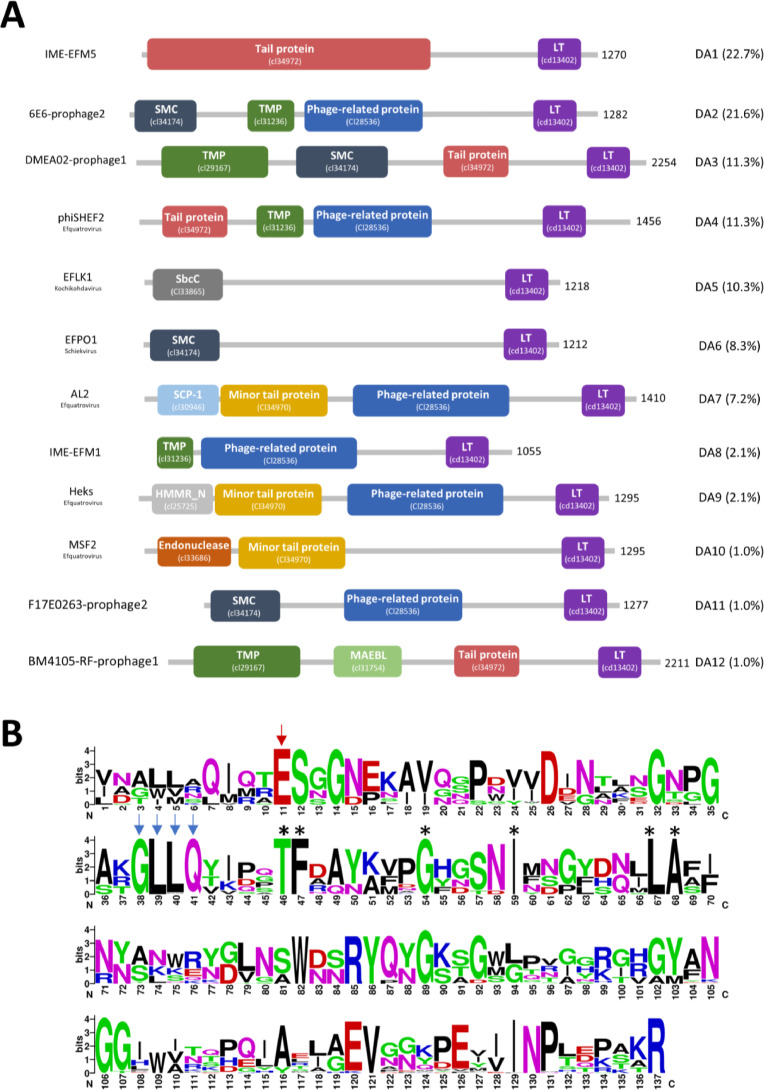
(A) Domain architecture of TMP-LT proteins. Based on CDD, 12 DA are shown with coloured domains. Purple= lytic transglycosylase-like domain (LT), Green= tape measure protein domain (TMP), Dark blue= structural maintenance of chromosomes (SMC), Red= Tail protein, Sky blue= Synaptonemal complex protein (SCP-1), Orange= Minor tail protein, Dark Grey=SbcC, Blue= Phage-related protein, Light green= Merozoite Apical Erythrocyte Binding-ligand (MAEBL), Brown= Endonuclease, light grey= Hyaluronan mediated motility receptor N-terminal (HMMR_N). The domain type and abundance (%) are indicated on the right side while phage or prophage name and the ICTV classification is on the left side. The asterisks indicate conserved residues specific for analysed sequences. The length of the protein is also mentioned. (B) Weblogo of the TMP-LT domains showing highly conserved domains including the catalytic residue Glutamic acid (red arrow) and GH23 specific motif (Blue arrows). Sequence logos were created using Weblogo (http://weblogo.berkeley.edu/logo.cgi).

Our next step of analysis analysed putative proteins containing Lytic transglycosylases (LT) domains, enzymes that degrade the peptidoglycan layer by cleaving the β−1,4-glycosidic bond between N-Acetylmuramic acid (MurNAc) and N-Acetyl-d-glucosamine (GlcNAc) (Holtje et al., 1975). Of the 98 LT detected, 97 are contained within putative Tail tape-measure proteins (TMPs) from the genomes of viruses with contractile or non-contractile tails. These contained a broad variety of predicted domain architectures but all had the LT domain at the C-terminal end (Fig. 6A). The TMP proteins are usually the longest proteins in the phage genomes (Piuri and Hatfull, 2006) and the predicted length here varied from 1180 to 2254aa (Fig. 6A). Moreover, our analysis found that the location of the TMP-LT proteins in predicted enterococcal siphovirus genomes is always the same (i.e. TMP-Dit-Tal) (Goulet et al., 2020). Other studies from phage infecting other species have also identified LT within the TMPs (Piuri and Hatfull, 2006; Stockdale et al., 2013). In our study, we do not see any other putative lysins than LT in TMP proteins.

Of note, the N-terminal region of the analysed TMPs often includes domains putatively involved in DNA binding or cleavage such as SCP-1,SMC,endonuclease and SbcC (Fig. 6A). This coincides with the putative proposed function of TMP as facilitating DNA delivery and injection into bacterial cells (Mahony et al., 2016).

Since lytic transglycosylases are carbohydrate targeting enzymes, the CAZy database was used to reveal that all the identified TMP-LT proteins belong to the specific glycosyl hydrolase (GH) family 23. The GH23 family includes lysozyme type G (EC 3.2.1.17), peptidoglycan lytic transglycosylase (EC 4.2.2.n1) and chitinase (EC 3.2.1.14). Amino acid sequence alignment and consensus analysis of our TMP-LTs revealed the presence of the GH23 conserved Glutamic acid (E) active site proton donor (Fig. 6B, red arrow). Previous studies assigned LT enzymes into 8 families based on sequence motifs (Dik et al., 2017). Our identified TMP-LT sequences shared motifs with family 1A: motif I includes the catalytic residue E-S, motif II contains the G-l-M-Q residues, motif III consists of A/G-Y-N residues and motif IV is a conserved Y residue flanked by a hydrophobic residue (Fig. S3A) (Dik et al., 2017). Indeed others have reviewed LTs and noted that the GXXQ of motif II is conserved amongst GH23 enzymes (Blackburn and Clarke, 2001; Dik et al., 2017; Scheurwater et al., 2008; Wohlkönig et al., 2010). Our data also revealed novel conserved motifs in the identified TMP-LT sequences that are not present in the family 1A i.e. T_46_, F_47,_ G_54,_I_59,_ L_67_, A_68_ (Fig. 5B). Of note, the enterococcal phage LTs examined here contain extra conserved residues not present in the LTs in the literature- from either other Gram positives, Gram negative bacteria or phage from Gram negatives. Hence, we propose a new family that we label 1P (for phage) (Fig. S3A).

Outside of the TMP-LTs discussed here, one outlier was found, this time in a predicted Studiervirinae genome EFA-2 (39.9 kb). EFA-2 has a large genome and an unusual genome organisation compared with the Sarlesvirinae podovirus genomes (Fig. 3B). However, the longest gene in this genome showed a predicted GH23-LT domain which is unusually located at the N-terminal end as opposed to the TMP-LTs which have the LT at the C-terminal end. CAZy database analysis showed that this LT has closest homology with Gram negative infecting phage lysins such as the *E. coli* T7 phage gp16 lytic transglycosylase protein (97% aa similarity) and is likely a member of family 1E as described by Dik et al. (2017) (Fig. S3B).

#### 3.3.4 Prophage pectinesterases potentially targeting EPA

After analysing the enterococcal phage and prophage genomes, seven predicted tail proteins from prophage, but none from lytic phage, were found to harbour a Tail-associated pectinesterase domain. Pectinesterases are enzymes that target pectin via demethylation of galacturonosyl residues (Reid, 1950). Pectin is a main component of plant cell walls and is made up of three types: a homopolymer of galacturonic acid, and two forms of a rhamnogalacturonan (RG-I and RG-II) made up repeating Gal-Rha disaccharides (Mohnen, 2008). Importantly, in enterococci, the cell wall contains a specialised polysaccharide called enterococcal polysaccharide antigen (EPA) that is made up of repeating rhamnose units, interspersed with other sugars and decorated with various modifications (Dale et al., 2017; Guerardel et al., 2020; Rigottier-Gois et al., 2015; Teng et al., 2009). Therefore, we hypothesise that the EPA structure in enterococci could be the target of these phage pectinesterases.

**Fig. 7:**
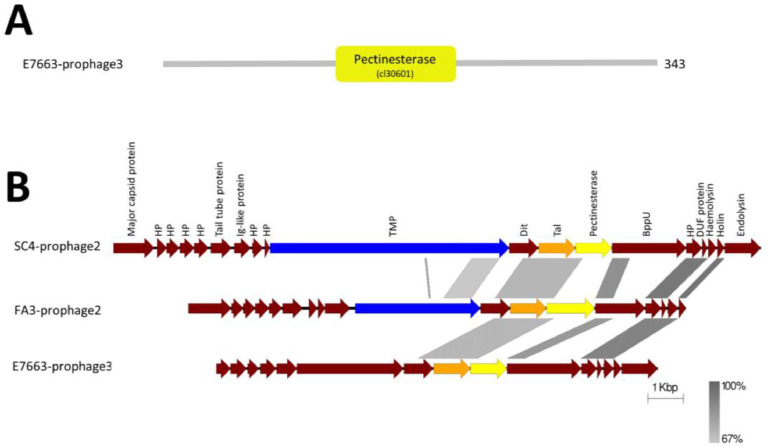
(A) Domain architecture of a Pectinesterase containing protein from E7663-prophage3 genome based on NCBI domain database which showed Pectinesterase domain. (B) MSA of tail modules showing Pectinesterase protein (yellow), TMP-LT (Blue), TAEP (Orange). HP= Hypothetical proteins. The level of identity is indicated by the grey region between genomes.

The pectinesterase domains in the seven sequences were located approximately in the central region of the proteins and no other predicted domains were identified (Fig. 7A). The pectinesterase genes were all located right after the common Tal position in siphovirus-type genomes (Efquatroviruses, Phifelviruses, Saphexavirus or Andrewesvirinae)(i.e. TMP-Dit-Tal) and of note were also present in concert with TAEP and TMP-LTs in 4 prophage genomes (Fig. 7B). To further confirm our annotation, structural homology using Phyre2 was performed which identified structural homologues in pectinesterase 1 or rhamnogalacturonan lyase families indicating that these putative genes may well be novel phage pectinesterases or EPA targeting enzymes. Similar pectinesterase/pectin lyase domains were also found in other phages targeting *Klebsiella pneumoniae* (Li et al., 2021; Pertics et al., 2021) and *Acinetobacter baumannii* (Shahed-Al-Mahmud et al., 2021) which all have showed a depolymerase activity upon expression and purification.

#### 3.3.5 Glycerophosphodiester phosphodiesterases (GDPD) often found in tail modules associated with TAEPs

**Fig. 8:**
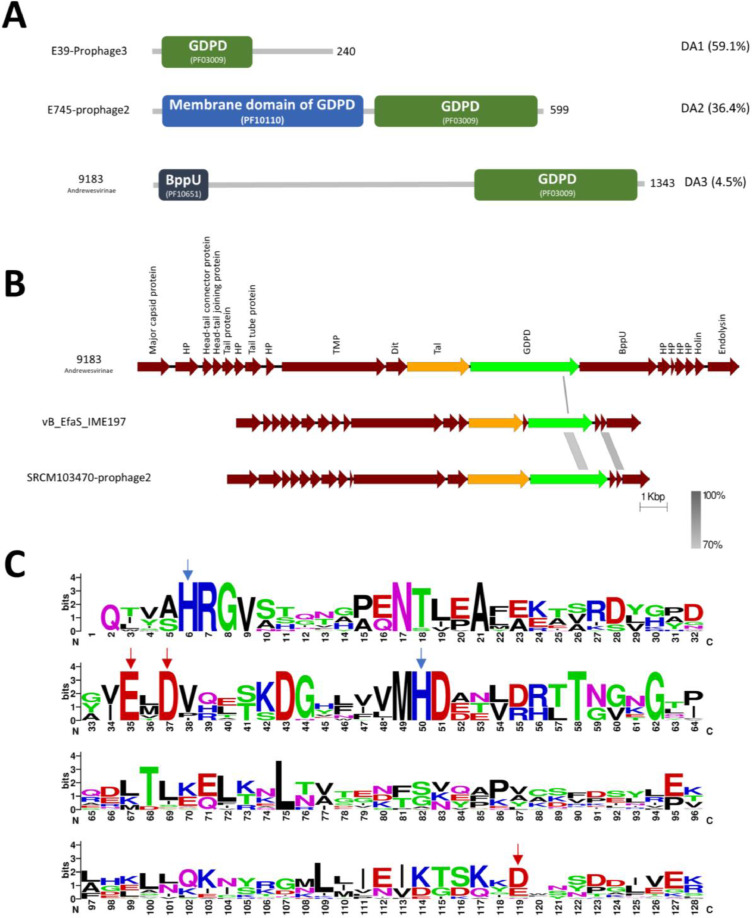
(A) Domains architectures of the GDPD containing proteins based on Pfam. Three DA are shown with coloured domains. Green= GDPD, Blue= membrane and dark blue=baseplate upper protein. The domain type and abundance (%) are indicated on the right side while phage or prophage name and the ICTV classification is on the left side. The length of the protein is also indicated. (B) MSA of tail modules showing GDPD protein (Green), TAEP (Orange), HP= Hypothetical proteins. The level of identity is indicated by the grey region between genomes. C) weblogo of the aligned GDPD domains which shows catalytic residues (Blue arrows) and metal binding residues (Red arrows).

The final type of predicted lysin observed are glycerophosphodiester phosphodiesterases (GDPD), confirmed using Pfam, NCBI domain database and Phyre2. These GDPD enzymes can degrade the phosphodiester bonds holding wall teichoic acids to sn-glycerol 3-phosphate (Gro3P) and their corresponding alcohol (Cornelissen et al., 2016). Our analysis revealed 22 gene predictions carrying the GDPD domain in both phage and prophage genomes. The GDPD proteins display three domain architectures (DA-PD)(Fig. 8A). The first and most common DA-PD (59.1%) contains only a GDPD domain (PF03009.17) with most sequences having a protein size of around 240aa. The second DA harbours a GDPD domain and a predicted membrane domain (PF10110.9) (Fig. 8A) homologues of which have found in *Streptococcus* bacterial genomes (Chuang et al., 2015). The third DA contains the GDPD domain at the C-terminus with a predicted baseplate upper protein (BppU) located at the N-terminal end indicating that this is likely a multifunctional baseplate-lyase protein in phage 9183. Some of these proteins were found within the tail module (e.g., phage 9183 (Andrewesvirinae), vB_EfaS_IME197, SRCM103470-prophage2 (Fig. 8B) while others were spotted throughout the genomes (e.g., BA17124, E39 and E745 prophages). It is also of note here that all the GDPD seen in the tail modules were in concert with a TAEP protein, suggesting potential synergy (Fig. 8B).

The GDPD activity of other phage encoded enzymes been investigated and showed that five conserved residues are required: 2 catalytic Histidines that act as a general acid and general base in catalysing the hydrolysis of the 3′−5′ phosphodiester bond (Rao et al., 2006; Shi et al., 2008) and 3 divalent metal-ion-binding residues (2 Glutamic acid residues and an Aspartic acid residue) (Cornelissen et al., 2016; Shi et al., 2008). In our analysis, the alignment of the enterococcal GDPD domains showed the presence of these highly conserved residues (Fig. 8C).

### 3.4 Common patterns in arrangement of lysins within genomes follow viral types

**Fig. 9:**
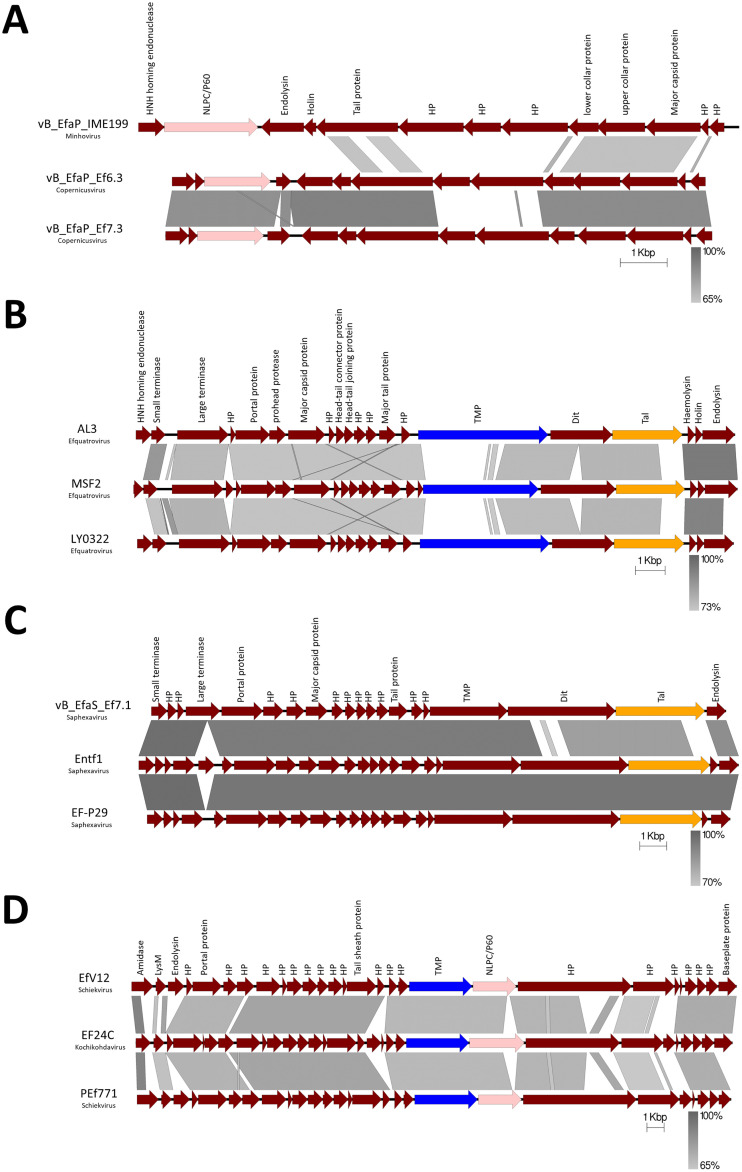
The general organisation of Tail modules in enterococcal phage genomes. (A) examples of podovirus genomes harbour NLPC/P60 containing protein (pink colour). (B) Group1 of siphovirus genomes contain both TAEP (orange) and TMP-LT (blue) proteins while Group2 (C) contains only TAEP proteins (orange). (D) myovirus genomes harbour TMP-LT and NLPC/P60 proteins. The phage names and the ICTV classification are indicated on the left side. The level of identity is indicated by the grey region between genomes.

During our analysis we noted several patterns in the arrangement of potential lysins within phage genomes and specifically their tail modules. For the siphoviruses, the tail proteins usually follow the TMP-Dit-Tal tail order (Goulet et al., 2020), and this is replicated in the enterococcal phage analysed here. Notably we observed that the type of lysin identified correlates with the phage genome size. Specifically, the smaller genome group (1, 21–43 kb, Efquatroviruses or Phifelviruses) contains TAEP proteins as well as Tape-measure LTs (TMP-LT) (Fig. 9B) while the larger siphoviruses (Saphexavirus or Andrewesvirinae) (55–86 kb) have only a single predicted protein with a lytic domain (TAEP) (Fig. 9C). Despite not identifying the Dit protein bioinformatically, in many cases we observed small Hypothetical proteins (HP) that we assume is the Dit protein in these phages. For the myoviruses (Schiekvirus or Kochikohdavirus), a TMP-LT protein and another adjacent tail protein containing an NLPC/P60 domain were identified in all analysed genomes (Fig. 9D).

Finally, enterococcal podoviruses (Copernicusvirus or Minhovirus), contain a morphology where the head seems to be connected to a baseplate with what one assumes is an infection system (and no tail measure protein is present). Hence, it is unsurprising that they do not display the TMP, Dit, Tal paradigm. Our data indicate that adjacent to the head and endolysin/holin pair most podoviruses genomes contain a predicted NLPC/P60 family protein (Fig. 9A). The location of this protein is highly conserved amongst the analysed podovirus genomes and is likely part of a potential tailspike protein (unpublished data, personal communication, GP Stafford).

As discussed earlier, we have observed a correlation between phage genome size and phage morphology (i.e. small genomes are usually podoviruses while larger genomes are myoviruses). However, the exceptions to this correlation such as the siphovirus phage EFRM31 (16.9 kb) showed no TAL while the siphovirus phage EFAP-1 (21.1 kb) contains both the TAEP and TALT proteins. For podoviruses, phage EFA-2 (39.9 kb) contains an LT containing protein (largest protein in the genome), which could also act similarly to a TMP.

Enterococcal prophages genomes seem to conform to the pattern of siphovirus (Efquatroviruses, Phifelviruses, Saphexavirus or Andrewesvirinae) type tail modules (TMP-Dit-Tal) (Fig. S4). For TAL, the majority (86.7%) of the prophage genomes have the endopeptidase TAEP in the Tal position, i.e. TMP-Dit-Tal(TAEP). The other genomic organisation observed is TMP with LT activity alone (6.9%). TMP and Tal with LT and TAEP activities, respectively, are observed in 6.3% of prophage genomes containing TAL (Fig. S4). Lastly, 59 of the prophage genomes did not contain predicted lysins associated with tail proteins and the functional modules in some of these genomes were not conventionally organised albeit they are predicted to be intact prophages by PHASTER.

Furthermore, we found that genome organisation within the enterococcal prophages coincides with that within isolated phages in terms of module order (i.e. Packaging, Head, Tail, Lysis, DNA Metabolism) (Fig. 3B). Additionally, the genome size of most analysed prophage was between 30 and 60 kb (Fig. 2B) and the majority possess the typical tail module arrangement seen in siphoviruses (Efquatroviruses, Phifelviruses or Saphexavirus) (i.e. TMP-Dit-Tal). As expected, we observed several lysogenic genes such as integrase, repressor and anti-repressor in these predicted prophages. Collectively, we propose that these prophages are likely to be Efquatroviruses, Phifelviruses or Saphexavirus.

## 4 Conclusion

In this study, we have surveyed the lysin landscape of enterococcal bacteriophage genomes. The most commonly identified TAL domains were those targeting peptidoglycan, namely endopeptidases (TAEP), NLPC/P60 (endo)peptidases as well TMP located GH23 lytic transglycosylases- present within tail-tape measure proteins in all predicted tailed viruses surveyed. Lastly other domains potentially target EPA (pectinesterases) and teichoic acids (GDPD). Overall, one predicts that these all target different parts of the cell wall of these enterococci and that the differences in domain and sequence indicate differences in strain specificity that are not yet elucidated. Additionally, the finding that many phages contain multiple potential lysin domains suggests a layer of co-operation between these domains *in vivo* that we have yet to elucidate.

Finally, our data reveal the extent and variety of enterococcal lytic domains as candidates for recombinant production as potential novel antimicrobials, either in isolation or in combination with each other or as potentiators of antibiotics. Finally, we have also laid a platform for potential engineering of enterococcal phage akin to the recent refactoring study on T7 (Liang et al., 2022). Our group are also currently working on expressing examples of a range of these genes recombinantly with a view to production of novel antimicrobials.

## Author statement

Manuscript: VIRUS-d-22–00,735

Both authors (AA and GS) carried out study conception and design, analysis and interpretation of data. They also drafted the manuscript and read and approved the final manuscript. Authors: Alhassan M Alrafaie (AA), Graham P Stafford (GS)

## Funding

This study is supported via funding from Prince Sattam bin Abdulaziz University project number (PSAU/2023/R/1444).

## Declaration of Competing Interest

The authors declare that they have no known competing financial interests or personal relationships that could have appeared to influence the work reported in this paper.

## Data Availability

Data will be made available on request.

## Appendix Supplementary materials

Image, application 1

Image, application 2

Image, application 3

Image, application 4
