# Supplementary material for: Enterococcal bacteriophage: A survey of the tail associated lysin landscape
Source: Virus Res. 2023 Feb 22;327:199073. doi: 10.1016/j.virusres.2023.199073 (PMC10194240; doi:10.1016/j.virusres.2023.199073)
Supplement: Supplementary file 4 [file mmc4.docx]

**Supplementary**

| 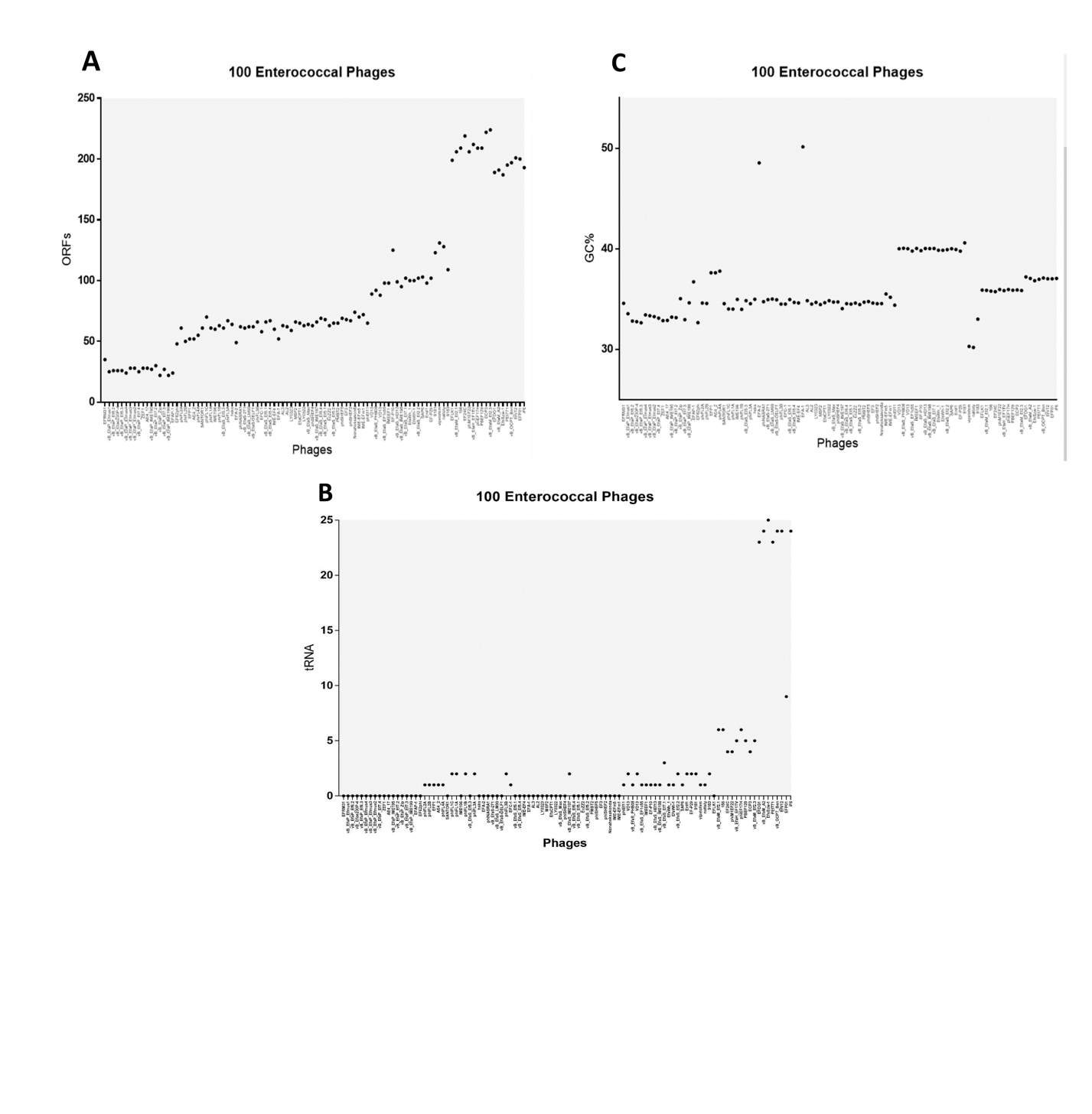  Fig. S1. 100 phage genomes were plotted against (A) ORFs, (B) tRNA and (C) GC%. The genomes are in ascending order in both Figures. |
| --- |

| 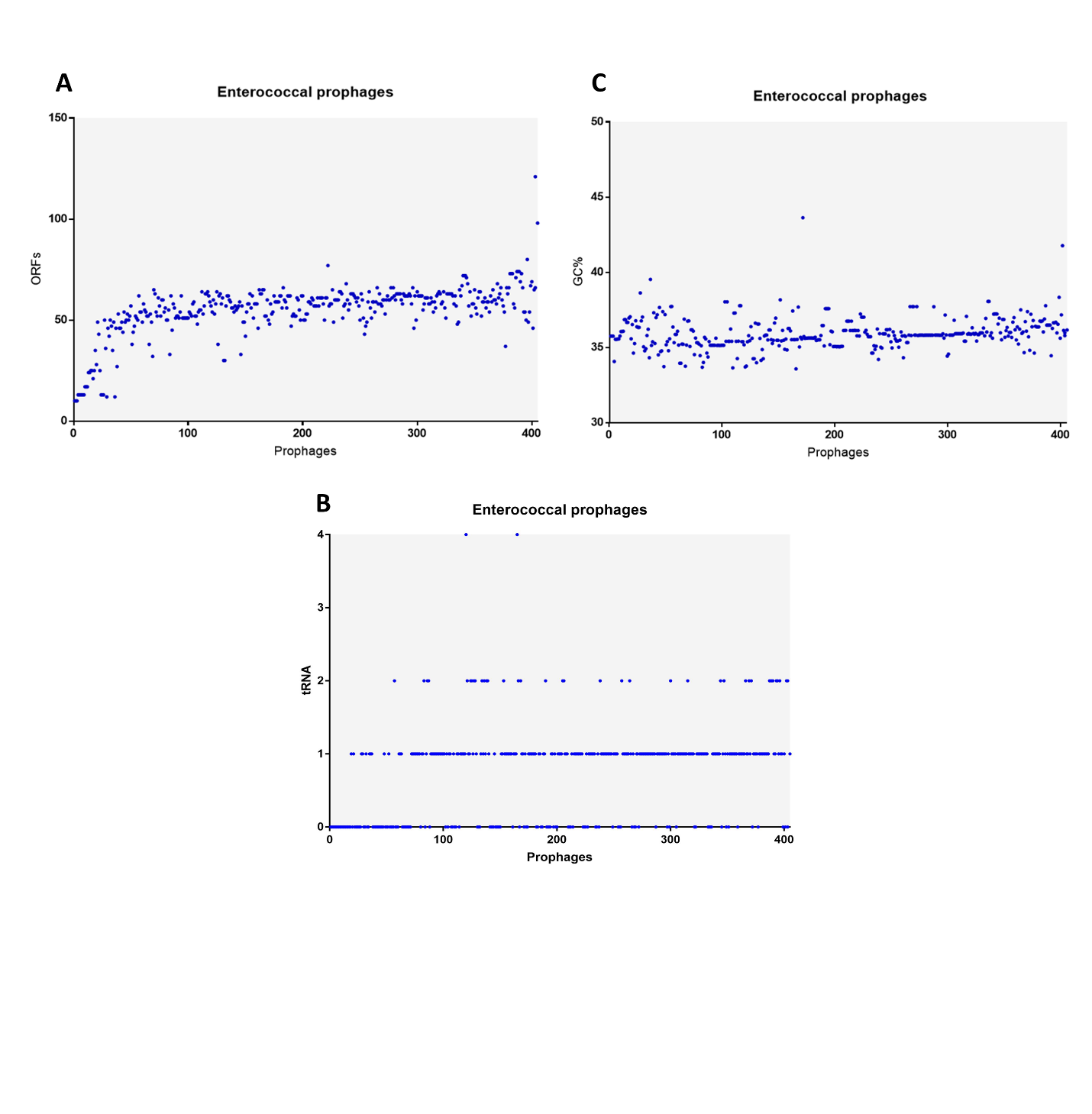  Fig. S2. 406 predicted prophage genomes were plotted against (A) ORFs, (B) tRNA and (C) GC%. The genomes are in ascending order in both Figures. |
| --- |

| 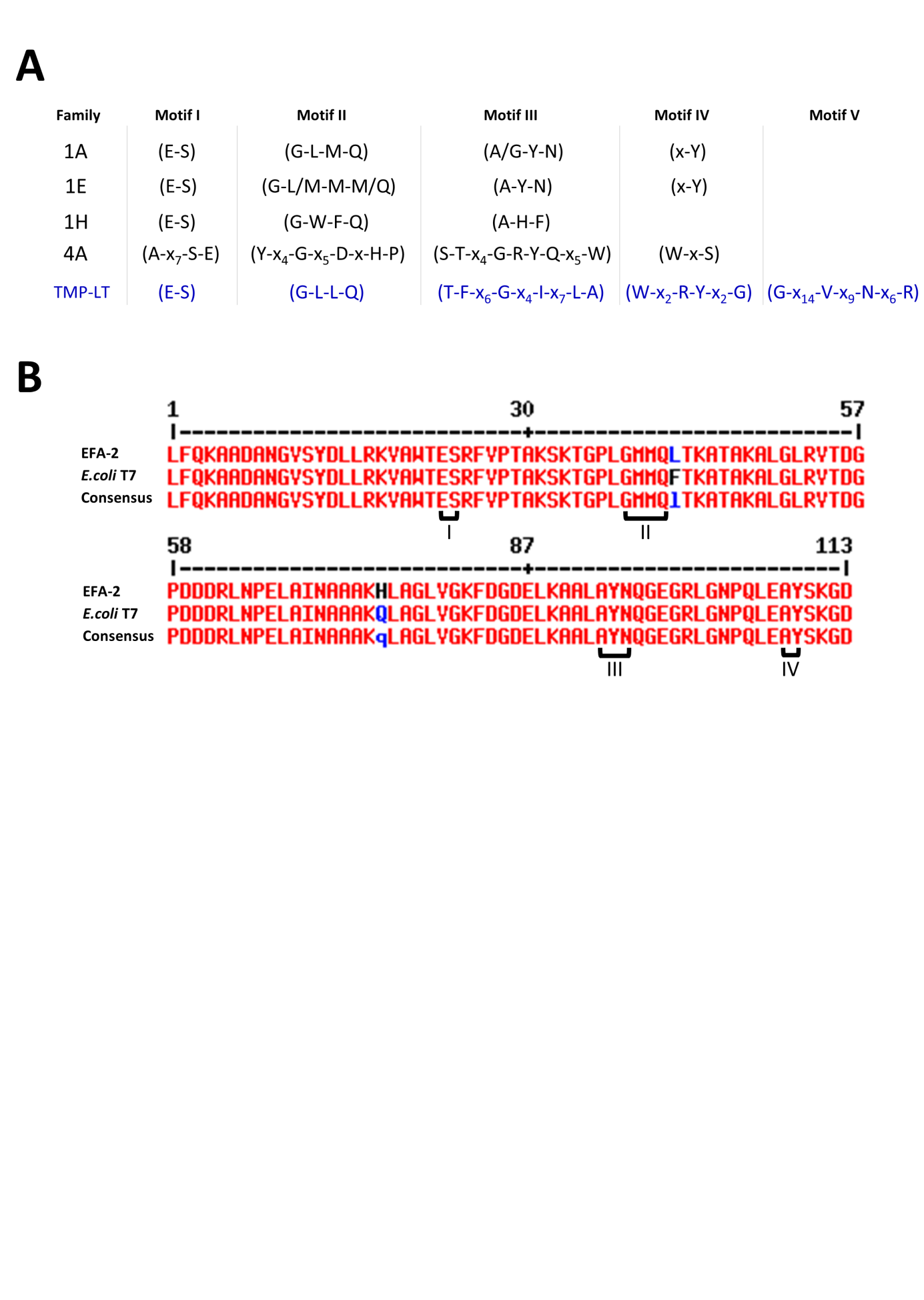  Fig. S3. A) The conserved motifs of families 1A,1E,1H,4A and TMP-LT (blue) are shown. B) Sequences alignment of LT domain between EFA-2 and *E.coli* T7 phages which motifs are numbered as I,II,III and IV. |
| --- |

| 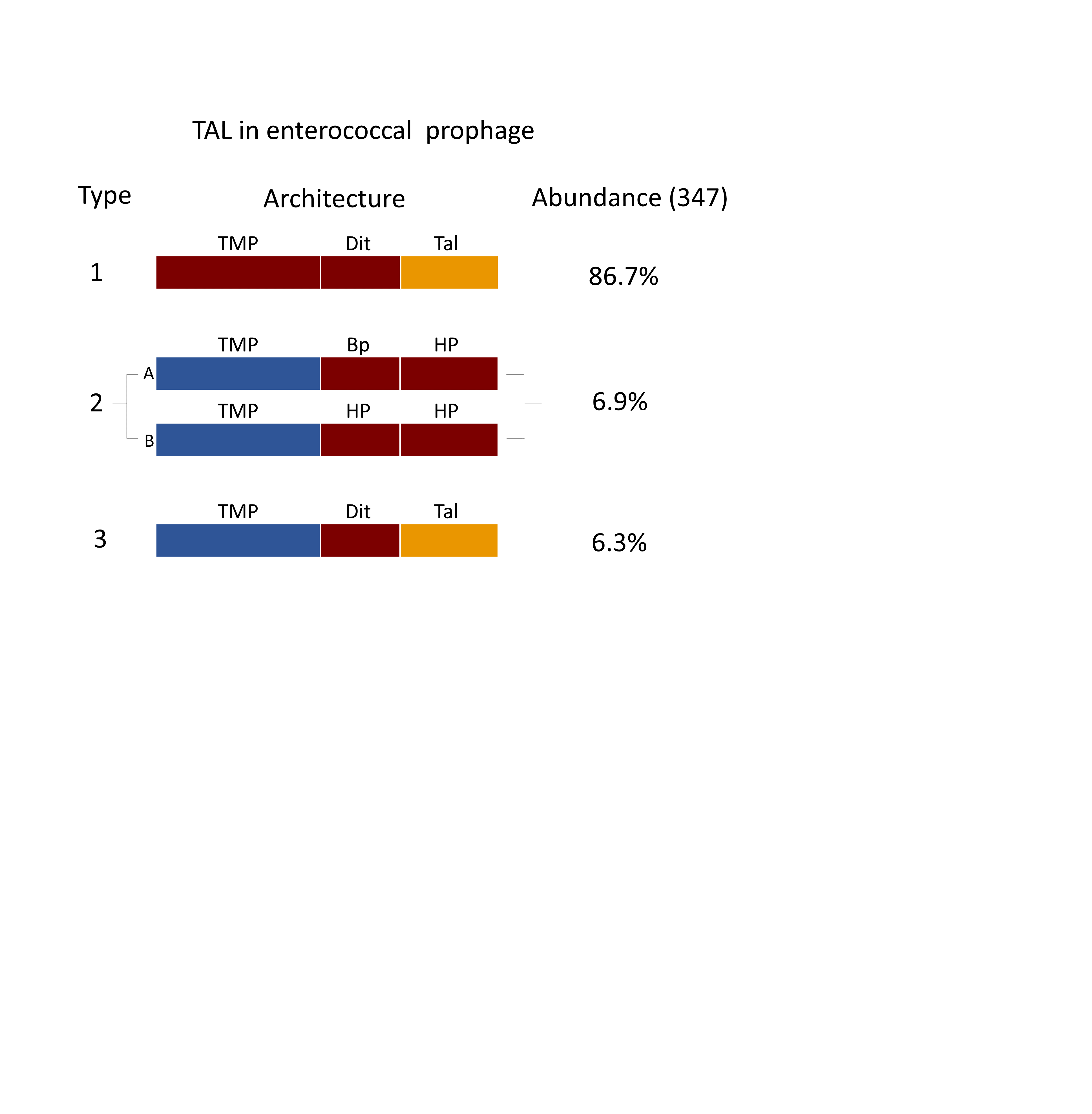  Fig. S4. TAL in enterococcal prophage genomes. Three main types are shown regarding the location of TAL in the tail module. The abundance in percentage is calculated from the total prophage genomes with TAL. Gene annotation is shown as follow: Tape measure protein (TMP), Tail-associated lysin (Tal), Baseplate protein (Bp), Hypothetical protein (HP), predicted endopeptidase activity (orange), LT (blue), No lysin activity identified (dark brown). |
| --- |
